# Predictive Factors for Pregnancy-Related Persistent Pelvic Girdle Pain (PPGP): A Systematic Review

**DOI:** 10.3390/medicina59122123

**Published:** 2023-12-05

**Authors:** Elisa Burani, Sharon Marruganti, Gloria Giglioni, Francesca Bonetti, Daniele Ceron, Alessandro Cozzi Lepri

**Affiliations:** 1 Musculoskeletal and Rheumatological Physiotherapy, Faculty of Medicine and Surgery, University of Rome Tor Vergata, 00142 Rome, Italy; elisaburani64@gmail.com (E.B.); sharon.marruganti@gmail.com (S.M.); gloria.giglioni1@gmail.com (G.G.); daniele.ceron@gmail.com (D.C.); 2 Private Practice “Kura” Clinic, 53047 Siena, Italy; 3 Asl Sud-Est, Department of Rehabilitation, Valdichiana Senese, 53045 Siena, Italy; 4 Asl Roma 3, Department of Rehabilitation, 00122 Rome, Italy; 5 Private Practice “Physioup” Clinic, 00142 Rome, Italy; 6 Private Practice “Riabilita” Clinic, 35030 Padova, Italy; 7 Medical Statistics and Epidemiology, Centre for Clinical Research, Epidemiology, Modelling and Evaluation (CREME), Institute for Global Health, London NW3 2PF, UK; a.cozzi-lepri@ucl.ac.uk

**Keywords:** persistent PGP, PGP postpartum, pregnancy-related PGP, predictive factors, systematic review

## Abstract

*Background and Objectives*: To identify the most frequently reported predictive factors for the persistency of pregnancy-related pelvic girdle pain (PPGP) at 3–6 months after childbirth in women with PPGP alone or PPGP in association with pregnancy-related lower back pain (PLBP). *Methods*: Eligibility criteria: Two authors independently selected studies excluding PPGP determined by a specific, traumatic, gynecological/urological cause or isolated PLBP and studies that did not include the presence/absence of PPGP as the the primary outcome. We, instead, included studies with an initial assessment in pregnancy (within 1 month of delivery) and with a follow-up of at least 3 months after delivery. *Data sources*: The research was performed using the databases of Medline, Cochrane, Pedro, Scopus, Web of Science and Cinahl from December 2018 to January 2022, following the indications of the PRISMA statement 2021 and the MOOSE checklist. It includes observational cohort studies in which data were often collected through prospective questionnaires (all in English). *Study appraisal and risk of bias*: Two independent authors performed evaluations of the risk of bias (ROB) using the quality in prognostic studies (QUIPS) tool. *Synthesis of results*: An in-depth qualitative analysis was conducted because, due to a high degree of heterogeneity in the data collection of the included studies and a lack of raw data suitable for quantitative analysis, it was not possible to carry out the originally planned meta-analyses for the subgroups. *Results*: The research process led to the inclusion of 10 articles which were evaluated using the QUIPS tool: 5 studies were evaluated as low ROB and 5 were evaluated as moderate ROB. High levels of pain in pregnancy, a large number of positive provocation tests, a history of lower back pain and lumbo-pelvic pain, high levels of disability in pregnancy, neurotic behavior and high levels of fear-avoidance belief were identified as strong predictors of long-term PPGP, while there was weak or contradictory evidence regarding predictions of emotional distress, catastrophizing and sleep disturbances. *Discussion*: The impossibility of carrying out the meta-analysis by subgroups suggests the need for further research with greater methodological rigor in the acquisition of measures based on an already existing PPGP core predictors/outcome sets.

## 1. Introduction

Pelvic girdle pain (PGP) identifies pain that arises in relation to pregnancy, trauma, arthritis or osteoarthritis; it is felt between the posterior iliac crest and the gluteal line, particularly near the sacroiliac joint. Pain may be referred down the thigh posteriorly and may or may not occur in association with symphysis pain [1]. It represents a multifactorial condition with a partly unknown etiology as it is essentially a biomechanical, traumatic, metabolic, genetic and degenerative factor [2]. PGP is a frequent condition during pregnancy (pregnancy-related pelvic girdle pain—PPGP); it can occur together with PLBP (pregnancy-related lower back pain) or, less frequently, separately. It has a prevalence which ranges between 23% and 65% of women, with some variability based on how the disorder is defined and measured [3]. Overall, 50% of pregnant women suffer from pregnancy-related lumbopelvic pain (PLPP) and, amongst them, 20% experience PPGP. Given the important heterogeneity of terminology in the literature, the incidence varies between 4% and 76%. Considering only the PPGP (excluding urological/gynecological causes) according to the definition of the “European Guidelines” of Cost Action 13, the incidence drops to 20%, with a high rate of misdiagnosis [4,5]. PPGP can occur in the first trimester of pregnancy (usually at the end), during delivery or in the first month postpartum [4]. The peak of symptoms generally occurs between the 24th and 36th week of gestation [6,7] and their localization may change during pregnancy [8,9,10]. Although 78% of women recover spontaneously 6 weeks after delivery [11], about 1/3 still show symptoms after 3 months and about 8.5% have important symptoms after 2 years.

It is not clear why only some women recover from PPGP and, for that reason, the identification of predictive factors for persistency would allow early targeting of women at risk of chronicity and the deployment of management strategies for modifiable risk factors.

## 2. Objective

The goal of this systematic review was to identify the most frequently reported modifiable predictive factors for the persistence of PGP 3 and 6 months postpartum in women with PPGP alone or with both PPGP and pregnancy-related LBP.

## 3. Methods

### 3.1. Study Selection

The eligibility criteria defined for articles’ selection are shown below.

Population: Articles concerning women with PGP or lumbo-pelvic pain or PGP and low back pain, regardless of their age, without stratification by number of births, or by type of birth, were included in the study.

Outcome assessment: Women were followed in prospective longitudinal observational studies, without therapeutic interventions, evaluated through self-reported questionnaires and/or clinical examination. Considering that the onset of PGP is located between the end of the first trimester of pregnancy and the first month postpartum (including the stage of labor), we have included studies with an initial assessment in pregnancy or within 1 month of delivery and with at least one follow-up visit at least 3 months after delivery.

Primary outcome: Presence/absence of PGP using the following definition of Vleeming, 2008: “PPGP identifies a pain that arises in relation to pregnancy, it is felt between the posterior iliac crest and the gluteal line, in particular near the sacroiliac joint. Pain may be referred down the thigh posteriorly and may or may not occur in association with symphysis pain”.

Secondary Outcome: Disability, depression, catastrophizing, quality of life, sleeping disorder.

Types of studies: Prospective observational cohorts and prospective questionnaires.

The following exclusion criteria have also been applied: 

Studies concerning specific PGP (inflammatory diseases, fractures, osteoporosis, neoplasia, other severe pathologies), traumatic PGP, PGP from gynecological/urological causes, pregnancy-related low back pain taken in isolation and studies that only investigated biological risk factors (e.g., BMI, hormone levels) were excluded, while studies in which both psychosocial-clinical presentation and biological factors were analyzed are included.

Failure to include the primary outcome presence/absence of PGP (VAS) was a criterion for exclusion of the studies; the same did not apply to secondary outcome measures which appeared heterogeneously in the studies.

*Information sources:* The search was performed by reviewing the literature on the Medline, Pedro, Cochrane, Scopus, Web of science and Cinahl databases from December 2018 to January 2022; only articles in English and published after 2000 were included.

*Search strategy:* The search string and keywords used were:

MEDLINE: (((“pelvic girdle pain”) OR “pelvic girdle pain”[MeSH Terms]) OR “pelvic girdle pain postpartum”) OR “pelvic girdle pain pregnancy-related”) AND ((“risk factors”) OR “risk factors”[MeSH Terms]): 51 papers.

PEDRO simple search: Pelvic girdle pain pregnancy: 34 papers (26 RCT, 8 SR).
COCHRANE: Pelvic girdle pain: 1 Cochrane protocol with 127 trials.
CINAHL ((pelvic girdle pain OR pelvic girdle pain pregnancy-related OR pelvic girdle pain postpartum)) AND (risk factors or contributing factors or predisposing factors): 150 papers.
SCOPUS (TITLE-ABS-KEY (pelvic AND girdle AND pain OR pelvic AND girdle AND pain AND pregnancy-related OR pelvic AND girdle AND pain AND postpartum) AND TITLE-ABS-KEY (risk AND factors)): 32 papers.
WEB OF SCIENCE ((pelvic girdle pain OR pelvic girdle pain pregnancy-related OR pelvic girdle pain postpartum)) AND (risk factors or contributing factors or predisposing factors): 158 papers.

**Figure 1:**
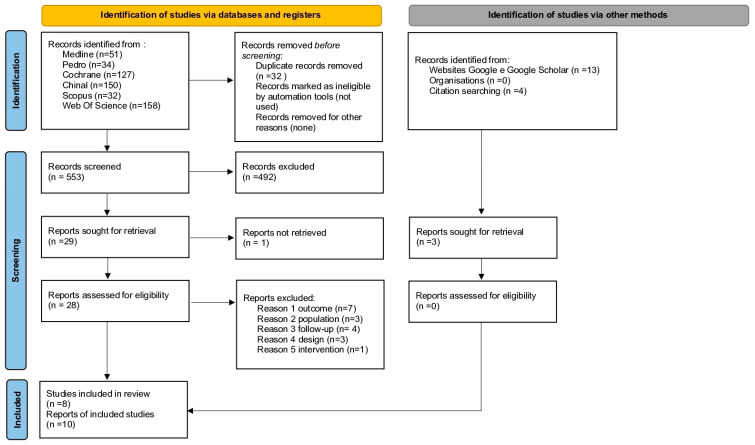
PRISMA 2020 flow diagram for systematic reviews which included searches of databases, registers and other sources [12].

The methodological process carried out by two independent authors (E.B. and S.M.), which led to the selection and inclusion of 10 articles is summarized in Figure 1 and Table 1; any disagreement between E.B. and S.M. was resolved through discussion with a third author (G.G.). The table with the characteristics, outcomes and other variables of the included studies is included in the supplementary material (Data S1).

### 3.2. Assessment of Risk of Bias

We analyzed the risk of bias (ROB) via evaluation by two independent authors using the quality in prognosis studies (QUIPS) tool and resolved discrepancies via discussion.

For the application of QUIPS we referred to Hayden A. and colleagues [38], while using the labelling suggested by Wuytack we assigned each item a “−” when it was evaluated as a low risk of bias, “+/−” as a moderate risk of bias and “+” as a high risk of bias.

The items considered are shown in Table 2. For the global assessment of the degree of risk of the individual studies (“overall”), we also referred to the Scottish Intercollegiate Guidelines Network (SIGN) for the evaluation of cohort studies which considers a high-quality study (low ROB) if the majority of the criteria are met, acceptable (moderate ROB) if most of the criteria are met, and low quality (high ROB) if many of the criteria are not met.

Slightly adapting these guidelines, we have given a low ROB to those studies in which 2 or fewer of the items were high/moderate ROB (i.e., had a “+” or a “+/−“ sign) and a moderate ROB to those studies in which 3 or more of items were high/moderate ROB (i.e., had a “+” or a “+/−“ sign). None of the studies were classified as a high ROB.

## 4. Data Synthesis

Among the articles included, it was possible to identify 3 main risk factor groups (pain intensity—VAS, previous LBP in pregnancy and number of positive provocation tests), but it was not possible to conduct a formal meta-analysis due to the heterogeneity of the outcome measures and the insufficiency of numerical data to enter in the quantitative calculations (see details in Data S3).

## 5. Results

### Study Selection and Characteristics

The 10 articles included in this systematic review are all prospective cohort studies. 

In 6 out of 10 studies, women were assessed both by questionnaire and by physical examination [15,19,24,25,27,35], while in 4 studies they were assessed only by questionnaire [16,20,22,33]. The primary outcome of all included studies was persistent PGP which was assessed as a binary outcome (presence/absence) in four studies [15,16,22,33] and by the quantitative measure VAS/NPRS in six studies [19,20,24,25,27,35]. The baseline outcome assessment was performed during pregnancy or within one month from delivery in all the included studies, while there was some variability for the duration of the follow-up (see Data S1). In nine studies, it covered the first 6 months and up to 24 months postpartum, while in one study the follow-up was up to 12 years [22].

The secondary analyzed outcomes were also heterogeneous, but in 5 out of 10 studies one of the secondary outcomes was disability, which was evaluated using various questionnaires: the Pelvic Girdle Questionnaire (PGQ) [19,25], the Disability Rating Index (DRI) [16,24] or the Oswestry Disability Index (*ODI*) [25]. Self-reported health status and quality of life were also evaluated as secondary outcomes in 5 out of 10 studies. Also, for the measurement of these responses we found heterogeneity in the questionnaires used: the Health Related Quality of Life (HR-Qol) [19], the EuroQol (EQ-5D) [25], the Short Form-36 Health Survey (SF-36) [27], the Self-Rated Health (SRH) [22] and the Nottingham Health Profile (NHP) [33].

The identified predictive factors were even more heterogeneous and therefore we decided to report the results separately for each study and attempted to form clusters of factors when they were sufficiently similar to each other (Table 3). We report below the main findings in each of the 10 papers.

Albert H. et al., 2001 [15] identified in a subset of women with PGS (n = 100) the group with the worst prognosis. Within these, they identified six factors correlated with the risk of persistence of pain at two years: (i) advanced age (≥29 years; RR = 1.9; *p* ≤ 0.05), (ii) poor education (RR = 2.3; *p* ≤ 0.05), (iii) non-qualifying work or unemployment (*p* ≤ 0.05), (iv) high pain intensity (VAS ≥ 6; RR = 1.6; *p* ≤ 0.05), (v) low test indices’ mobility (≤320; RR = 3.9; *p* ≤ 0.005) and (vi) a high number of positive provocation tests (≥16; RR = 10.7; *p* ≤ 0.001).

Beales D.J. et al., 2018 [19], followed a group of 29 women on average at 15 months postpartum (SD = 2.0) who had low-to-moderate levels of disability and pain at baseline (PGQ = 28, SD = 26; NRS = 2, SD = 3) and that in 41% of cases (n = 12) reported continuous pain at follow-up. Three characteristics (ASLR performance, sleep quality through PSQI and PPT in five parts of the body) at baseline and three at 15 months postpartum: pain intensity (NRS), pain quality (Mc Gill), disability (PGQ) and quality of life (SF-36), were evaluated. After performing a Spearman correlation analysis, the authors found that a poor performance in ASLR during pregnancy was correlated with a low quality of life at 15 months postpartum (*Spearman rho* = −0.558, *p* < 0.05, the exact *p*-value was not reported) and that a reduced PPT at the level of the sacrum during pregnancy was correlated with high McGill scores (*rho* = −0.384, *p* < 0.05). 

According to Bergström C. et al., 2014 [20], women with a history of LBP before delivery were 2.47 times more likely to report “recurrent pain” (OR = 2.47; *p*-value = 0.03) and 3.35 times more likely to report “continuous pain” (OR = 3.35; *p*-value = 0.02) at follow-up at 14 months postpartum compared to those who at 14 months had had a remission of symptoms (“no pain”); the presence of LBP before pregnancy was found to be a strong predictor of pain 12–14 months postpartum.

High levels of pain during pregnancy and in the first six months postpartum were also associated with a worse outcome at 14 months after delivery: this finding is in contrast with those reported in the paper by Olsson, 2012 [16], which does not identify this as a risk factor for long-term LPP.

For Bergström C. et al., 2017 [22], the most important predictor of a poor outcome for women with PPGP at 12 years seems to be “wide spread pain”: indeed, the authors found a statistically significant correlation with the presence of sciatica (OR = 3.4 (95% CI:1.87–6.20); *p* < 0.0001) and neck/thoracic pain (NP/TP) (OR = 2.50 (95% CI:1.40–4.48); *p* = 0.002). The presence of NP/TP for more than 30 days in the last 12 months was associated with 2-fold higher odds of developing the event (OR = 2.03 (95% CI:1.06–3.87); *p*-value = 0.03). Furthermore there is a strong correlation between the presence of LBP and long-term PPGP (OR = 2.50 (95% CI:1.40–4.48); *p*-value = 0.002) and the correlation between disability and PPGP (OR = 4.03 (95% CI:0.87–18.73); *p*-value = 0.08).

According to Robinson H. et al., 2010 [39], women who showed 3–4 painful areas during physical examination in pregnancy (predicted difference in mean pain intensity = 18.7 (95% CUI: 7.9, 29.6); *p*-value = 0.007) or more than 6–8 positive provocation tests (mean difference (md) = 11.2 (95% CI:2.4, 19.8); *p*-value = 0.04) had higher pain levels at 12 weeks postpartum. A pre-pregnancy BMI of ≥25 kg/m^2^ (md = 5.7 (95% CI: −0.3, 11.8, 11.8); *p*-value = 0.05) was associated with the intensity of pain, but the result was only borderline statistically significant.

The presence of pre-pregnancy LBP (md = 5.0 (95% CI:0.5–9.5); *p*-value = 0.03) and 6-8 positive provocation tests on physical examination (md = 7.7 (95% CI:1.1–14.3); *p*-value = 0.03) was associated with DRI at 12 weeks postpartum. When using “non-recovery at 12 weeks” as the dependent variable in a logistic regression model, the authors found that the number of painful sites (OR = 4.4 (95% CI:1.3–14.6); *p*-value = 0.02) and the sum of positive provocation tests (OR = 3.5 (95% CI:1.2–10.3); *p*-value = 0.02) were associated with the risk of non-recovery at 12 weeks, while pre-pregnancy BMI was only weakly associated (OR = 2.1, *p*-value = 0.05). According to Gausel A. et al., 2015 [25], the combination of three independent risk factors (age ≥ 30 years (OR = 2.9 (1.3–6.8); *p*-value = 0.012), moderate/high ODI in pregnancy (OR = 5.1 (1.7–15.0); *p*-value = 0.003) and PP with LBP in pregnancy (OR = 2.8 (1.2–6.4); *p*-value = 0.017) was associated with a risk of developing persistent PGP which was 27 times higher than the risk in women with none of these factors, with an absolute risk (AR) of 35%.

Robinson H. et al., 2014 [27], investigated pain and disability in women with PGP at the 30th week of gestation and with one-year follow-up. Twelve weeks after delivery there was no evidence for a difference in outcomes according to the considered variables (pain localization, ASLR, P4 and PGP at the 30th week of gestation) except for the symphysis which correlated with higher levels of ache.

By one year from delivery, there was no evidence for a difference in the average level of disability, while women who reported PGP at 30 weeks of pregnancy, and who had pelvic pain, or who had positive P4 and ASLR were found to have a higher degree of pain.

Olsson C. et al., 2012 [16] identified six predictors of persistent lumbo-pelvic pain pregnancy related to 6 months postpartum: (i) catastrophizing (PCS), (ii) avoidance behaviors (FABQ), (iii) intensity of current pain and of (iv) worst perceived pain (VAS), (v) disability (DRI) and (vi) quality of life (NHP). The presence of catastrophizing, which was considered as an exposure in this analysis (HR = 2.05, 95% CI:1.06–3.98; *p*-value = 0.034) and disability (HR = 2.29, 95% CI:1.10–4.47; *p*-value = 0.026) at 19–21 weeks of gestation were shown to be independently associated with a higher risk of postpartum LPP. 

Fernando et al., 2020 [33] confirms that high levels of fear-avoidance beliefs (FABQ) at 34–37 weeks of pregnancy can lead to a higher risk of having persistent low back pain at 6 months after delivery with an OR of 1.06 (95% CI:1.01–1.12, *p = 0.03*). According to Xiangsheng et al., 2021 [35], after controlling for confounding factors, high levels of neurosis assessed with the quick big five personality test (QBFPT) were associated with persistent PGP after pregnancy (OR = 2.03, 95% CI:1.92–2.13) *p = 0.002*), while extroverted and conscientious behaviors tended to show a protective effect against the disorder (OR = 0.79 95% CI:0.71–0.87, *p* = 0.004; OR = 0.92, 95% CI:0.87–0.97, *p = 0.021*, respectively). 

## 6. Risk of Bias of Included Studies

The 10 included studies were qualitatively assessed using the QUIPS tool. During the evaluation with the QUIPS tool, 5 studies were overall classified as low ROB and 5 studies as moderate ROB.

The final results of the ROB assessment are shown in Table 2, while in the Supplementary Materials (Data S2) there is the evaluation of the articles with the specifications for each domain considered by the QUIPS tool.

## 7. Synthesis of Results

**Figure 2:**
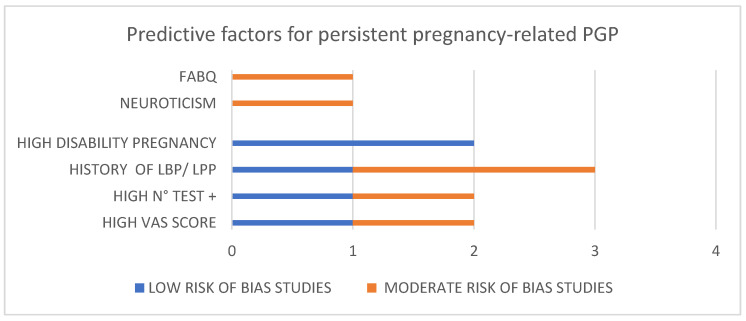
Numbers of studies that analyzed the main predictive factors and respective level of ROB (risk of bias).

Our review shows that the most investigated factors which were also significantly correlated with the risk of long-term PGP analyzed in the included studies are: (i) high levels of pain in pregnancy—VAS score (Albert, 2001—moderate ROB; and Bergström, 2014—low ROB), (ii) high number of positive provocation tests (Albert, 2001—moderate ROB; Robinson, 2010—low ROB), (iii) LBP/LPP history (Bergström, 2014—moderate ROB; Robinson, 2010—moderate ROB; Gausel, 2015—low ROB), (iv) high levels of disability in pregnancy (Gausel, 2015—low ROB; Olsson, 2012—low ROB), (v) neuroticism (Xiangsheng, 2021—moderate ROB) and (vi) high levels of FABQ (Fernando, 2020—moderate ROB) (Table 3, Figure 2).

## 8. Principal Findings

In our review, a history of LBP was shown to be associated with the risk of persistent PGP in the largest number of studies (Bergström, 2014 [20], Robinson, 2010 [24], Gausel, 2015 [25]) with a magnitude of the effect which was >2-fold (OR = 2.47; OR = 23 and OR = 4.4, respectively) and significative *p*-values (<0.05), although two of these studies were rated as moderate ROB (Bergström, 2014 [20], Robinson, 2010 [24]). The intensity of pain (VAS scale) and the number of positive provocation tests have been analyzed in two studies with variable quality (one low ROB, one moderate ROB), with an estimate of the relative risk ranging between 1 and 3 (*p*-value < 0.05). However, different tests are typically used to evaluate pain during pregnancy and it remains to be established which of these is the most useful to identify women with the highest risk.

Two studies which we classified as low ROB (Gausel, 2015 [25] and Olsson, 2012 [16]) could lead us to conclude that the presence of disability in pregnancy seems to predispose to the persistence of PGP at 6 months after delivery (respectively, OR = 5.2; HR = 2.14, *p*-value < 0.05). In our review, three studies rated as low ROB [16,22,25] and three rated as moderate ROB [24,33,35] evaluated the psychosocial domains and found that neuroticism (OR = 2.12; *p*-value = 0.001) and fear-avoidance beliefs were associated with the risk of persistence of PGP. In particular, according to Fakari F.R. et al., 2018, FABQ scores tended to vary with pain severity [40] and according to Fernando, 2020, high FABQ scores at 34–37 weeks of gestation were predictive of PPGP with an OR = 1.06; (*p*-value = 0.03) [33]. In contrast, the presence of emotional distress and depression in pregnancy were not found to be associated with either pain or postpartum disability; although depressive symptoms were shown to be three times more frequent in women with LPP [17,41,42], the study by Gausel, 2015 [25] could not identify a cause–effect relationship. The same limitation applies to most of the studies included in this review, including the association between levels of catastrophization and PPGP in the long term (Olsson 2012 [16]).

Among other possible risk factors evaluated, of note, it was found that unskilled work, more than the workload itself, was associated with the risk of persistence of pain at 24 months after childbirth. Van den Berg, 2012 [14] shows a correlation between maintaining uncomfortable postures at work, mainly intended as positions with repeated twists and bends, and the risk of PGP at the 30th week of pregnancy and 6 weeks after delivery. Pre-pregnancy BMI appears to be associated with both disability and pain 3 months after delivery in one study, although the result was not statistically significant; Bjelland [23] and Matsuda [43], however, confirmed that a BMI > 30 in pregnancy was associated with persistent PGP at 6 months after delivery.

On the contrary, characteristics of childbirth, the number of children, the number of pregnancies, the type of pregnancy [17], the weight and sex of the child, marital status, the use of contraceptives or other hormonal treatments, urinary infections in the years prior to pregnancy and the presence of diastasis of the rectus abdominis [44] were not correlated with the risk of persistence of pain in any of the included studies.

## 9. Comparison with Existing Literature

Overall we identified in Data S1 approximately 15 different predictive factors (unskilled work, education, level of pain (VAS), mobility index, number of positive test, ASL test, pressure pain threshold (PPT), previous LBP, self-rated health (SRH), sick leave, widespread pain, disability, catastrophization, fear avoidance, neurosis), of which only 6 were deemed to be important on the basis of their frequency of reporting, effect size measures, strength of the association and quality of studies (ROB) (see Table 2 and Table 3, Figure 2).

Our results are consistent and extend those previously reported in the literature and those shown in a similar previous systematic review. For example, in Clinton’s guidelines in 2017, the early onset of pain, localization of pain in several points, high number of positive provocation tests, dissatisfaction at work and low expectation of recovery are reported as the main determinants of the persistence of PGP in “late pregnancy” and postpartum [45]. In particular, the high intensity of pain and the number of painful sites have been identified as important factors in the transition from acute pain to chronic pain and persistent disability [23,46,47]. Regarding the psychological domains, high levels of emotional distress [2,16,23,24,48,49], catastrophizing [16,18,37] and the patient’s poor expectation of recovery were also identified as potential factors for persistence [48].

Our review identifies four additional risk factors: (i) history of LPP, (ii) high level of disability in pregnancy, (iii) fear-avoidance beliefs level and (iv) neurotic behavior. These findings suggest that the collection of clinical information and the study of psychosocial factors in pregnant women is key, as the ultimate aim is to reduce the risk of long-term PPGP.

Additional factors such as sleep disorder, neck–thoracic pain, headache, fibromyalgia and pain characteristics are considered important for the persistence of PPGP [14,50,51] and for most of them no anatomical abnormalities or specific inflammatory or degenerative processes can be identified through diagnostic tests [52]. Despite the growing number of studies correlating central sensitization patterns with sleep disturbances [41,48,52], it is still a poorly investigated factor for PGP. Only 1 (Beales, 2018) [19] of the 10 studies included in our review detected a possible association with PPGP in agreement with a previous study of the same author (Beales, 2016) [51] showing an association between disorders of sleep and PGP in pregnancy, but not with persistent PPGP.

## 10. Strengths and Limitations

Some limitations need to be mentioned before drawing firm conclusions. These include a bibliographic search performed only in English, a limited number of studies given by rather restrictive inclusion/exclusion criteria and the strong heterogeneity of outcome measures’ acquisition in the included studies [53], which prevented the performance of a formal quantitative meta-analysis. In particular, confounding factors were not adequately controlled for or reported in all studies which makes the comparisons even more difficult.

Given the small number of papers dealing with the specific topic and the variable methodological quality [54], our study by means of an updated overview of the main predictive factors suggests that an improvement in the quality of longitudinal studies is warranted which should be based on the already existing standardized PPGP classification system and core outcome/predictors’ evaluation sets [53,55].

## 11. Conclusions and Implication

Given the large number of identified predictive factors, the heterogeneity of outcome assessment, methods to control for confounding and the length of follow-up in the included studies, it was not possible to conduct a quantitative analysis. Consequently, it is difficult to draw strong conclusions on which are the most important factors to predict persistent PPGP. Although we held prior knowledge about modifiable predictive factors of PPGP, this review specifically points at six key factors that should be investigated by the clinician during pregnancy: high pain intensity, high number of positive provocation test, history of LPP, high disability, neurotic behavior and a high fear-avoidance beliefs level (see infographic in Data S4). Some of these had been missed in previous similar work. Nevertheless, it remains to be established which tests are more useful to carry out during pregnancy and whether would be helpful, for example, to adopt a self-screening battery test in early pregnancy as proposed by Olsen in 2014. However, further studies are needed to evaluate the reproducibility and reliability of the latter approach [56].

The processes underlying the development of postpartum long-term pain and disability are different from those identified for the onset of PGP in pregnancy [26] and this complexity means that it is necessary to frame the woman in early pregnancy, according to biomechanical, psychosocial and neurophysiological factors. This type of management should allow chronicity processes’ prevention, aimed at reducing individual women’s pain and concomitantly lowering the costs for society [39,57,58].

## Figures and Tables

**Table 1:** List of screened articles and reason for exclusion.

Articles Included or Excluded	Inclusion	Exclusion Reason
Sjodahl, 2013 [13]	No	Lack of primary outcome (VAS). Baseline time to follow-up does not satisfy our criteria.
Van den Berg, 2012 [14]	No	Population from clinical trial.
Albert, 2001 [15]	Yes	
Olsson, 2012 A [16]	No	Primary outcome: catastrophization.
Eisenach, 2008 [17]	No	First follow-up 8 weeks postpartum.
Vollestad, 2009 [18]	No	Population from clinical trial.
Elden, 2016 [2]	No	Population from clinical trial. Baseline time to follow-up not clear.
Beales, 2018 [19]	Yes	
Bergström, 2014 [20]	Yes	
Bergström, 2016 [21]	No	Primary outcomes do not satisfy our criteria.
Bergström, 2017 [22]	Yes	
Bjelland, 2012 [23]	No	Primary outcome evaluated like numbers of painful points.
Robinson, 2010 B [24]	Yes	
Gausel, 2015 [25]	Yes	
Olsson, 2012 B [16]	Yes	
Robinson, 2010 A [26]	No	Lack of postpartum follow-up.
Robinson, 2014 C [27]	Yes	
Gausel, 2020 [11]	No	Follow-up does not satisfy our criteria.
Ceprnja, 2021 [28]	No	Cross-sectional design.
Lindgren, 2014 [29]	No	Unclear terminology (back pain).
Bergström, 2019 [30]	No	Cross-sectional design.
Lardon, 2018 [31]	No	Primary and secondary outcomes do not satisfy our criteria.
Rost, 2006 [32]	No	Therapeutical intervention.
Fernando, 2020 [33]	Yes	
Munro, 2017 [34]	No	Primary outcome body pain, not specific for PPGP. Full text in French.
Xiangsheng, 2021 [35]	Yes	
Kovacs, 2012 [36]	No	Cross-sectional design.
Bakker, 2013 [37]	No	Lack of postpartum follow-up.

**Table 2:** Assessment of the risk of bias of the included studies.

Assessment Risk of bias Included Articles through QUIPS Tool							
Articles	Study Participation	Study Attrition	Predictive Factor Measurement	Outcome Measurement	Study Confounding	Statistical Analysis and Reporting	Overall
Albert, 2001 [15]	−	−	+/−	−	+	+/−	Moderate risk of bias
Beales, 2018 [19]	−	−	−	−	+	−	Low risk of bias
Bergström, 2014 [20]	−	+	+/−	−	+/−	−	Moderate risk of bias
Bergström, 2017 [22]	−	−	−	−	+	−	Low risk of bias
Robinson, 2010 [24]	+/−	+	−	−	+	−	Moderate risk of bias
Gausel, 2015 [25]	−	+	−	−	+	−	Low risk of bias
Olsson, 2012 [16]	−	+/−	−	−	−	−	Low risk of bias
Robinson, 2014 [27]	−	−	+/−	−	+/−	−	Low risk of bias
Fernando, 2020 [33]	+/−	+	−	+/−	−	−	Moderate risk of bias
Xiangsheng, 2021 [35]	+/−	+	−	−	+/−	−	Moderate risk of bias

+ High risk of bias, +/− moderate risk of bias, − low risk of bias.

**Table 3:** Clusters of factors associated with PPGP long-term.

Prognostic Factor	OR/RR	*p* Value	References
**Pain**			
High pain (VAS ≥ 6)	RR = 1.6	*p* < 0.05	Albert, 2001 [15]
High sacral PPT	RR = 3.24	*p = 0.008 (personal communication)*	Bergström, 2014 [20]
Widespread pain	Spearman rho = −0.384	*p* = 0.040	Beales, 2018 [19]
	OR = 2.03	*p* = 0.03	
**Provocation tests**			
High number of +test	RR = 10.7 (>16 + response)	*p* < 0.001	Albert, 2001 [15]
	OR = 5.0 (6–8 + test)	*p* = 0.002	Robinson, 2010 [24]
**Disability**			
High pregnancy disability			
	OR = 4.03	*p* = 0.08	Bergström, 2017 [22]
	OR = 5.2	*p* < 0.002	Gausel, 2015 [25]
	HR = 2.14	*p* = 0.072	Olsson, 2012 [16]
**LPP/NP/TP**			
	OR = 2.47	*p* < 0.030	Bergström, 2014 [20]
History LPP	Spearman rho = 0.09	*p* < 0.05	Robinson, 2010 [24]
	OR = 2.8	*p* = 0.017	Gausel, 2015 [25]
History NP/TP			
	OR = 2.50	*p = 0.002*	Bergström, 2017 [22]
**Fear**			
High fear avoidance	OR = 1.06	*p* = 0.03	Fernando, 2020 [33]
**Behavior**			
Neuroticism	OR = 2.03	*p* < 0.001	Xiangsheng, 2021 [35]

OR = odds ratio; RR = risk ratio; HR = hazard ratio. PPT = pressure pain threshold; LPP = lumbo-pelvic pain; LBP = low-back pain; NP = neck pain; TP = thoracic pain; PPGP = pregnancy-related pelvic girdle pain.

## Data Availability

Data sharing not applicable.

## Supplementary Materials

The following supporting information can be downloaded at: https://www.mdpi.com/article/10.3390/medicina59122123/s1, Data S1: Papers’ characteristics, outcome and other variables. Data S2: Evaluation of risk of bias (QUIPS tool). Data S3: Meta-analysis proof. Data S4: Infographic of 6 predictive factors.

## Registration

PROSPERO registered 5.7.2020 CRD4202018583. Review status: Review ongoing.

## Review Protocol

The protocol can be accessed at https://www.crd.york.ac.uk/prospero/display_record.php?ID=CRD42020185831 (accessed on 1 October 2023).

## Abbreviations

PGP	Pelvic Girdle Pain
PPGP	Pregnancy-Related Pelvic Girdle Pain
PLB	Pregnancy-Related Low Back Pain
LPP	Lumbopelvic Pain
PGS	Pelvic Girdle Syndrome
SIJ	Sacroiliac Joints
LDL	Long Dorsal Sacroiliac Ligament
P4	Posterior Pelvic Pain Provocation Test
ASLR	Active Straight Leg Raise
BMI	Body Mass Index
VAS	Visual Analogic Scale
PPT	Pain Pressure Threshold
PMI	Pregnancy Mobility Index
PSS	Perceived Stress Scale
DRI	Disability Rating Index
ODI	Oswestry Disability Index
PGQ	Pelvic Girdle Questionnaire
HR-Qol	Health Related Quality of Life
EQ-5D	EuroQol
SF-36	Short Form-36 Health Survey
SRH	Self-Rated Health
NHP	Nottingham Health Profile
PCS	Pain Catastrophizing Scale
PSQI	Pittsburgh Sleep Quality Index
FABQ	Fear-Avoidance Beliefs Questionnaire
NHP	Nottingham Health Profile
McGill	McGill Pain Questionnaire
NP	Neck Pain
TC	Thoracic Pain
ROB	Risk of Bias
QUIPS	Quality In Prognosis Studies Tool
OR	Odd Ratio
RR	Relative Risk
HR	Hazard Ratio
CI	Confidence Interval
