# Supplementary material for: Predictive Factors for Pregnancy-Related Persistent Pelvic Girdle Pain (PPGP): A Systematic Review
Source: Medicina (Kaunas). 2023 Dec 5;59(12):2123. doi: 10.3390/medicina59122123 (PMC10744457; doi:10.3390/medicina59122123)

Supplementary materials

Data S1: papers characteristics, outcome and other variables

Papers	Study Design	Population	Outcome	Outcome measures	Evaluation time	Investigated predictive factors	Predictive factors showing correlation
**Albert, 2001 [16]**	Epidemiological prospective cohort study	Pregnant women with pelvic pain, divided into 5 groups according to classification (33 weeks of pregnancy); evaluated with: questionnaire + physical exam (SJI provocative test; hip mobilidy), both performed from T0 to T6.	Persistent PGP at 2 years	Presence/absence	T0= 33 week of pregnancy gest.<br>T1= 1 month after delivery<br>T2=3 months after delivery<br>T3=6 months after delivery<br>T4= 12 months after delivery<br>T5=18 months after delivery<br>T6=***24 months*** after delivery	Age<br><br>Education<br><br>Job<br><br>Pain (VAS)<br><br>Mobility<br><br>Number of positive provocative test	Factors - of persistence after two years:<br>old age<br><br>unskilled work<br><br>poor education<br><br>high pain intensity (VAS)<br><br>low scores on the mobility index<br><br>number of positive provocative tests (factors present in the pelvic girdle syndrome-worst prognosis group)
**Beales, 2018 [23]**	Prospective cohort study, exploratory	Cohort of pregnant women from previous cross-sectional (pregnant women who were healthy between the 2-3 trimester of pregnancy).<br><br>Evaluate with: questionnaire + physical exam (ASLR, algometer). Both performed at T0 and T1.	Intensity of LLP<br><br>Quality of the pain<br><br>Disability<br><br>Quality of life with respect to pain	Intensity of pain: NPRS<br><br>Quality of pain: Mc Gill Pain Questionnaire<br><br>Disability: Pelvic Girdle questionnaire<br><br>Quality of life: HRQol(36-item short form health survey)	T1= 2-3 quarter of pregnancy<br><br>T3= 11-***18 months*** postpartum	Performance ASLR during pregnancy<br><br>Quality of the sleep (Pittsburgh Sleep Quality Index)<br><br>Pressure pain thresholds (PPTs)	Poor performance at ASLR correlates with reduced HRQol pp but not with pain intensity, pain quality, or disability.<br><br>Low PPT correlates with high pain quality scores.

**Bergstrom, 2014 [17]**	Prospective cohort study	Pregnant women who developed PLBP and PGP (cross-sectional population given Q1) and who showed persistent pain 6 months after delivery (these women make up the cohort).<br><br>Evaluate with: questionnaire.	Primary outcome:<br><br>PLBP and PGP at 12 months after delivery<br><br>Secondary outcomes:<br><br>Pain status<br><br>Health status<br><br>Family situation	Prevalence of PLBP and PGP (VAS)<br><br>Pain status (VAS)<br><br>Health status Self-rated health (SRH)<br><br>Family situation	T0= right after delivery<br><br>T1= 6 months after delivery<br><br>T2= ***12 months*** after delivery.<br><br>In the study he names T0 as T1 and the others accordingly.<br><br>The cohort includes only women who showed pain 6 months after delivery.<br>The questionnaire at T0 describes the gynecological history, current pregnancy and childbirth.	Pain at the moment of Q1<br><br>Persistence of PLBP and PGP postpartum<br><br>Number of children<br><br>Physical activity (yes/no, days per week, days between delivery and starting again activity)<br><br>Pain (VAS for intensity; descriptive variables for the type of pain; localization in front, behind, both)<br><br>SRH (before, during and after pregnancy) (very good, good)<br><br>Family situation (very good, good, etc)	Previous episodes of LBP<br><br>High levels of pain in pregnancy and in the first 6 months postpartum<br><br>Poor SRH in subjects with continuous pain during pregnancy and six months after delivery compared with the "recurrent pain" group
**Bergstrom, 2017 [24]**	Cohort study with follow up at 12 years	Women 12 years postpartum who had reported PGP during pregnancy (cohort of women recruited in 2002)<br><br>Evaluate with: questionnaire at T0, T1, T2, T3.	Persistent PGP at 12 years after delivery	Presence/absence (pain/no pain)	T0= right after delivery<br>T1=6 months postpartum<br>T2= 12 months postpartum<br>T3= ***12 years*** postpartum<br><br>The study name T0 as Q1 and the others accordingly.	Age<br><br>Neck pain/thoracic spinal pain in the last 12 months (+ the number of days where pain was experienced)<br><br>Days with PPGP in the last 12 months<br><br>Sciatica<br><br>Recovery expectations on an NRS<br><br>Sick leave in the last 12 months<br>Sick leave days in the last 12 months<br><br>Disability pension for PGP<br><br>Treatments required for PGP since the last pregnancy	Presence of more than 30 days of PPGP in the last 12 months<br><br>Sick leave for PGP in the past 12 months<br><br>Regional spinal pain (widespread pain) for more than 30 days in the last 12 months.

						Prescription / non-prescription drugs<br><br>Regular physical activity since the last pregnancy<br>BMI<br><br>Self-perceived health status (SRH)<br><br>Smoke<br><br>Alcohol consumption (AUDIT)<br><br>Marital status<br><br>Satisfaction in sex life	
**Robinson, 2010<br>B<br>[29]**	Prospective cohort study	Pregnant women with PGP in late pregnancy (evaluated up to 12 weeks postpartum)<br><br>Evaluate with: questionnaire + physical exam (ASLR, P4, distraction test, compression test, Patrick-Faber test, pubic symphysis palpation and LDL)	Persistent PGP<br><br>Disability	Intensity of pain (VAS)<br><br>DRI disability	T0=pregnant, at the first contact with the maternity center (questionnaire)<br><br>T1= 30 weeks of pregnancy (questionnarie+physical exam)<br><br>T2= ***12 weeks*** postpartum (questionnaire)	Evaluated for inclusion:<br><br>Age<br>Number of children<br><br>Family status<br>Education<br><br>Smoke<br><br>Pre-pregnancy physical activity<br><br>Pre-pregnancy BMI<br><br>Evaluated at 30 weeks:<br><br>Distress (HSCL-25)<br><br>Fear avoidance beliefs (mFABQ)<br><br>Localization of pain<br><br>Pain in other parts of the body<br><br>LBP history<br><br>ASLR<br>P4<br>Distraction test<br>Compression test<br>Patrick/faber test	Correlation with disability:<br>LBP pre pregnancy<br>Sum of positive provocative tests (BMI pre-pregnancy, ASLR associated but not statistically significant)<br><br>Correlation with pain intensity: number of painful sites, sum of positive provocative tests (BMI pre-pregnancy associated but not statistically significant).

						Palpation of the pubic symphysis<br>Palpation of the LDL	
**Gausel, 2015 [27]**	Epidemiological prospective cohort study	Women who had PP during pregnancy (population taken from a previous retrospective study).<br><br>Evaluate with: questionnaire + physical exam (walking, neurological exam, Patrick-Faber test, LDL palpation, Gaenslen's test, modified Trendelemburg, ASLR)	Persistent PGP (and how it affects life activities) it actually evaluates also:<br><br>disability<br>ADL<br>Health status	Presence/absence of PP (NRS)<br><br>ODI<br><br>PGQ<br><br>EQ-5D	T0= 1 day pospartum (questionnaire)<br><br>T1-T2 =3-***6 months*** postpartum (treats it as a time span). Questionnaire+ physical exam only for women who previously shown persistent PGP)	Age<br><br>Primiparous<br><br>Schooling<br><br>Heavy workload<br><br>BMI before pregnancy<br><br>Depression during pregnancy<br><br>Physical activity before pregnancy<br><br>Physical activity during pregnancy<br><br>PP and/or LBB 1 year before pregnancy<br><br>PP and/or LBP in pregnancy<br><br>ODI in pregnancy	Age> = 30 years<br><br>Score at the ODI moderate/high during pregnancy<br><br>History of PP and LBP in pregnancy
**Olsson, 2012 b [25]**	Prospective study	Pregnant women with and without LPP (19-21 week of pregnancy) followed up to 6 weeks postpartum.<br><br>Evaluate with: questionnaire (the same between T0 and T1).	Persistent PGP	Presence/absence (yes/no question)	T0=19-21 weeks of pregnancy<br><br>T1= ***6 months*** postpartum	Catastrophizing (PCS = Pain Catastrophizing Scale)<br><br>Beliefs on how physical activity affects back pain (FABQ = Fear Avoidance Beliefs Questionnaire)<br><br>Pain intensity (VAS) assessed at the present and worst time<br><br>Disability (DRI = Disability Rating Index)<br><br>Quality of life (NHP = Nottingham Health Profile)<br>Some background factors were evaluated which were considered significant if associated with the outcome:<br>Age<br>Marital status	Catastrophizing (PCS)<br><br>Disability (DRI)

						Occupation<br>Physical activity<br>Number of previous pregnancies<br>Sick leave<br>Reason for sick leave<br>Caesarean section<br>Onset, frequency, location of any LPP	
**Robinson, 2014 C [28]**	Prospective cohort study	Pregnant women with PGP at 30 week during pregnancy. Followed up to 1 year postpartum.<br><br>Evaluate with: questionnaire + physical exam (ASLR, P4)	Persistent PGP<br><br>Prevalence of PGP at 1 year after delivery<br><br>Clinical course of PGP from 30 weeks of pregnancy to 1 year after delivery<br><br>Disability<br><br>Pain	Presence/absence of PGP (yes/no)<br><br>SF-36<br><br>VAS (both for disability and pain)	T0=15 weeks of pregnancy<br><br>T1= 30 weeks of pregnancy (questionnaire + physical exam)<br><br>T2= 12 weels postpartum (questionnaire + physycal exam)<br><br>T3= ***1 year*** postpartum (questionnaire)	Presence of PGP at 30 weeks<br><br>Localization of pain in the pelvis<br><br>Response to clinical tests (P4 and ASLR)<br><br>They also collected some demographic factors:<br>age<br>education<br>marital status<br>smoke<br>job<br>number of children	The unfavorable clinical course does not appear to depend on these factors
**Fernando, 2020 [22]**	Prospective cohort study	Pregnant women (470) between 19 and 21 weeks with or without LP, from 3 clinics.	Presence/ absence of self-report of LP at 6 months	Self-report questionnair e yes/no Complete LP from 260 women	T1=19-21 weeks of pregnancy<br><br>T2=34-37 weeks of pregnancy<br><br>T3=***6 months*** postpartum	Pain level (VAS)<br><br>Physical Ability and Disability (DRI)<br><br>Health-related quality of life (HRQL)<br><br>General health status (NHP> distress in 6 areas: emotional reactions, sleep, energy, pain, physical mobility, social isolation)<br><br>Catastrophizing (PCS)	High levels of fear avoidance beliefs at 34-37 weeks show correlation with the risk of having LP at 6 months postpartum.
**Xiangsheng, 2021 [21]**	Prospective study	387 women at 12 weeks of pregnancy, who had PGP during the last 4 weeks, classified into no PGP (123) and PGP group (264)	Presence/ absence of PPGP	VAS>3 for more than 1 week	T1= 12 weeks of pregnancy<br><br>T2=6 months postpartum<br><br>T3= ***2 years*** postpartum	QBFPT:<br><br>outgoing<br>conscientious<br>neurotic<br>open to experiences<br>easy going	High levels of neurosis are associated (OR=2.12) with persistent PGP after delivery.

Note: maximum follow-up duration in bold for each study.

Data S2*:* evaluation of risk of bias (QUIPS tool).

Evaluation of Risk of Bias through QUIPS Tool of the 10 included papers					
Papers	Study participation	Study attrition	Prognostic Factor Measurement	Outcome Measurement	Study Confounding
Albert, 2001	a. +/-<br>amongst all the evalued women (1789) 405 were eligible<br>b.-<br>c.-<br>d. -<br>e.+/-<br>(place ok, time interval not specified)<br>f.– (adequately reported even if in a confusing way for the reader)	a.–<br>(15,8% drop-out)<br>b.–<br>c.–<br>(it is said that of the 64 drop outs, they have no info abut 18 and therefore no follow-up)<br>d.+<br>(the description of the 18 is not present and among the dropouts we find subjects without pain and therefore influencing the prognosis in the negative)<br>e.–	a.–<br>b.+/-<br>(some instruments are objectified, others subjective and therefore at risk of bias)<br>c.–<br>(indicates the cut off for pain and for the mobility index)<br>d.-<br>e.+<br>(the data of the analyzed group are complete but those of the other groups are not present)<br>f.+<br>(no methods of imputation are described)	a.+/-<br>(outcome is defined even if is an unclear way)<br>b. –<br>(the presence/absence is evalued but VAS is not used even if for the design of our study the presence is enough)<br>c.-	a.+<br>(is declared that not any confounding factors have not been measured)<br>b.+<br>c.+<br>d.+<br>e.+<br>f.+<br>g.+
Beales, 2018	a. + (sample of patients at baseline small - 39 women then reduced to 29)<br>b. –	a. –<br>(there are no drop-outs but it does not clearly state it anyway)	a. –<br>b. –<br>c. -<br>d.-<br>e. -	a. –<br>b. –<br>c. –	a. + (it is declared that they have been measured only at baseline and not at follow-up)

	c. –<br>d. –<br>e. –<br>f. –<br><br>Notes: although the sample is small, this item is clearly satisfied in all its points	b. NV for the absence of drop-out<br>c. – (6 lost (15,4 %)+ 4 excluded on criteria)<br>d. – (the characteristics of the lost to follow-up are reported)<br>e. – (it is stated that there are no differences between the lost and the included)	f. NV no data were lost		b. +<br>c.+<br>d.+<br>e. + (it is not carried out because a sample is too small)<br>g. +<br>Notes: within the limits of the study they declare that they have measured confounding factors such as psychological ones at the baseline and not at the follow-up but that they have not carried out the multivariate analysis due to the sample being too small
Bergstrom, 2014	a. –<br>b. –<br>c. +/- (The doubt was about the description of the characteristics of the participants in Q1 and Q2, but actually they consider Q3 as the study group, whose they give the characteristics at the baseline even if only of the 176 who respond)<br>d. –<br>e. –<br>f. +/- (partly exposed but could have been more specific and clearer)	a. – (27,3%: this percentage is obtained from the 639 women who were affected in Q1 who were administered Q2 464 respond)<br>b. + (it just says they do not answer)<br>c. + (no reasons are given: 12% follow-up because the other women were excluded because they were unaffected)<br>d. + (it only says that they do not respond but does not make a description of them to understand their characteristics)<br>e. – (it declares that there are no differences except for the age at the first birth)	a. +/- (fa lot of confusion is made between the variables)<br>b. –<br>c. –<br>d. +/- (sIt seems so, but in the measurement of risk factors it seems to me that there is little clarity)<br>e. –<br>f. NV	a. –<br>b. –<br>c. -	a. –<br>b. + (they are measured but not clearly stated as confounding factors)<br>c. –<br>d. –<br>e. NV<br>f. +<br>g. +<br><br>Notes: the last two items in my opinion have a high risk of bias because these factors are considered but not as real confounding factors and for example there is no stratification or multivariate analysis to evaluate the influence of factors on the outcome
Bergstrom, 2017	a. – (624 because they did not know the address of 15)<br>b. –	a. – (2,3 % of drop-out address change/missing answers)<br>b. – (they explaind	a. –<br>b. –<br>c. –<br>d. –<br>e. –<br>f. NV	a. –<br>b.- (VAS is not used but it is however indicative for the purpose of our study)<br>c. -	a. – (they are measured)<br>b. + (they are not defined as confounding factors)<br>c. +

	c. – (baseline description at Q4)<br>d. –<br>e. –<br>f. +/- (they are deduced but are not clearly spelled out)	addresses were missing)<br>c. +/- (of 624 administred questionnaire, we have answer from 295 47%. The item is partially satisfied because of 16 they say they refuse to participate and do not give consent and the other 313 only say that they do not answer)<br>d. – (writes a paragraph for the description of non-respondents)<br>e. +/- (there are differences in terms of number of children, age, marital status, alcohol consumption even if at baseline there are no significant differences)			d. +<br>e. NV<br>f. +<br>g. +<br>A multivariate regression is done but not for the confounding factors which however are not declared as such
Robinson, 2010 (B)	a. – (326)<br>b. –<br>c. +/- (incomplete: in the description of the cohort it defines some characteristics at the baseline and also in the description of the questionnaire at inclusion but subsequently in the table defines the characteristics of the 179 women who answer the questionnaire at the 12th week but we do not have the baseline data of the whole cohort)<br>d. – (describes both the administration of the questionnaire and to whom , and the	a. – (3,6% of drop out 3 drop out and 9 spontaneous abortions)<br>b. + (does not describes the drop-outs)<br>c. +/- (Apparently there are not losses at follow up but actually of 31 women we only have the questionnaire at 30th week without physical exam, and they are not counted in the final 179 finali so I count 9% of loss at follow up)<br>d. + (does not define why 31 women do not pass through physical exam)<br>e. + (not described and therefore the	a. – (also clearly listed for the evaluation method)<br>b. –<br>c. – (see statistical analysis from "we divided pre-pregnancy BMI)<br>d. +/- (9%-31 women are only subjected to a questionnaire without physical examination)<br>e. –<br>f. + (no methods of imputation of lost data are used because the 31 women are excluded from the final count)	a. –<br>b. –<br>c. -	a. - (They are not declared as confounding factors but are treated as risk factors but nevertheless they are measured)<br>b. +<br>c. +<br>d. +<br>e. NV<br>f. +<br>g. +

	peformed physical exam)<br>e. +/- (the recruitment period is missing)<br>f. + (can be inferred from the way the study was conducted but the inclusion and exclusion criteria were not clearly defined)	differences with the others are not described either)			
Gausel, 2015	a. – (541 interviewed women 330)<br>b. –(it describes they come from a previous retrospective study)<br>c.+ (I do not find the characteristics of the sample at the baseline; in the text it says that they are measured but the data is not reported / something is briefly described in the inclusion process)<br>d. –<br>e. –<br>f. +/- (they are deductible but unclear)	a. – (19 + 9 drop out-4,92%)<br>b. + (it is only said that they do not answer)<br>c. + (21 lost at follow-up (30%) reason for loss at follow up is not described it is only said that some do not want to be examined)<br>d. + (the lost participants are not described and therefore do not allow for the fulfillment of criterion e.)<br>e. -/+ (the results assume the presence of persistent PGP similarly between the 21 who dropped out and those who participated)	a. –<br>b. –<br>c. –<br>d. +/- (some women do not undergo a physical exam but are given a questionnaire)<br>e. +/- (21 women do not undergo physical examination)<br>f. – (data for women not subjected to physical examination are calculated based on the rest of the population)	a. –<br>b. –<br>c. +/- (21 women do not undergo clinical exam)	a. - (they are measured)<br>b. + (they are measured but are not declared a priori)<br>c. -<br>d. –<br>e. + (they are measured but are not declared and are measured as prognostic factors but not as confounding factors and therefore the multivariate analysis is not carried out)<br>f. + (they are measured but are not declared and are measured as prognostic factors but not as confounding factors and therefore the multivariate analysis is not carried out<br>g.+ (they are measured but are not declared and are measured as prognostic factors but not as confounding factors and therefore the multivariate analysis is not carried out)
Olsson, 2012 (B)	a. -<br>b. –<br>c. –<br>d. –<br>e. +/- (the place but not the time period of	a. +/- (31 % of drop out)<br>b. – (Drop out are described in the flowchart)<br>c. – (16% lost to follow-up are	a. –<br>b. –<br>c. –<br>d. –<br>e. +/-(it is stated that there are low internal missing	a. –<br>b. – (LPP it is measured only as presence/absence and not through the VAS but for the purpose of our	a. –<br>b. –<br>c.-<br>d. –<br>e. NV (no missing confounder data)<br>f. –

	recruitment is described)<br>f. +/- (defines to include both women with and without LPP but does not establish other inclusion / exclusion criteria)	described in flowchart)<br>d. +<br>e. + (nNo differences are described and it is not stated that there are no differences between the lost and the included)	values except for the FABQ-act)<br>f. + (it is not declared the use of appropriate imputation methods for missing data)	study the presence/absence is sufficient)<br>c. -	g.-
Robinson, 2014 (C)	a. – (326 at the beginning)<br>b. –<br>c. – (table 1 and description in the text)<br>d. -<br>e. –<br>f. + (it only says it excludes 18 women in a new pregnancy but does not clearly define the exclusion and inclusion criteria)	a. – (13% of drop out: I considered as drop out all the lost before the 30th week even if in the text only 3 drop outs are described)<br>b. – (in fig. 1 only the reasons are described; in my opinion, when it comes to drop-outs, those lost without motivation are indicated)<br>c. – (reasons in figure 1 and when drop-outs are discussed, in my opinion it refers to those without reasons- 7% lost at first follow-up and 18,2 % lost at the second follow up) in the total dropout calculation, 47 are considered because the others with motivation are spontaneous abortions or new pregnancies)<br>d. -<br>e. +/-(at the beginning of the results, the total cohort is compared with the sample study, highlighting differences that are not significant for our study except in part whether they are used or not)	a. -<br>b. -<br>c. – (in statistical analysis from "study women were categorised")<br>d. +/-(at 1 year the physical examination is not performed and therefore, as there have been losses at the follow-up, the evaluation method is not the same for all participants)<br>e. +/- (the data is shown only for 215 and only in the graph unclear)<br>f. +/- (it is unclear but only the 215 data are reported)	a. -<br>b.-<br>c.-	a. – (they are measured)<br>b. + (they are not declared as confounding factors)<br>c. -<br>d. -<br>e. NV there are not lost data<br>f. +/-<br>g. +/- (for f and g items they are adapted only for age)

Fernando, 2020	a.+/- Characteristics described in another study<br>b.+/- 5 clinics of 3 demographic areas, precise characteristics in other studies<br>c.-<br>d.+/- it does not say which clinics and which geographic areas<br>e.+ it does not specify inclusion/exclusion criteria<br>f.+/- there is no list of features at the baseline	a.+/- response rate almost halved<br>b.+<br>c.- motivated in flow chart too<br>d.+ characteristics of the lost are not reported<br>e.+we don't know wheter there was difference	a.-<br>b.-<br>c.-<br>d.+ they complete it at home<br>e.-<br>f.+ imputation for missing is not identified	a.-<br>b.+/- self evaluation<br>c.+ home	a.-<br>b.-<br>c.+/-<br>d.-<br>e.+ missing data<br>f.-<br>g.-
Xiangsheng, 2021	a.+/-<br>b.+/- it calculates sample size<br>c.+/- Can not be understood if the time frame is referring to the study or to the recruitmenr<br>d.+/- tertiary hospital antenatal clinic<br>e.- well expressed criteria<br>f.- well described, also the flow chart	a. +/- 100% declared response rate (?)<br>b.+ o N/A<br>c.+ o N/A<br>d.+ o N/A<br>e.+ o N/A	a.-<br>b.-<br>c.-<br>d.+/- partly in the clinic partly at home data collection<br>e.-<br>f.? missing is not declared	a.- also in flow chart<br>b.-<br>c. +/- interview or phone interview	a.+/- it is said that counfonders are considered, but it is not stated which<br>b.+ the most incident is not specified<br>c.+/- reported in tab 3 but not in full<br>d.+/- unknown<br>e.+ missing are not considered<br>f.- comparable groups<br>g.+/- not all the confounding factors are considered
+ represents high risk of bias, +/- represents moderate risk of bias, - represents low risk of bias (individual item assessment)					

Data S3: *meta-analysis proof and graphic study of correlation*

Author	Year	N° total	No PGP	Incidence	Risk Factor	ev/exp	ev/noexp	noev/exp	noev/noexp	OR/RR raw	95% CI	BMI OR/RR adjusted	95% CI	Confounders	Notes
Albert	2001	341	29	8,5	VAS>=6 (pain during pregnancy)					1,6					describe media VAS score , no %
Bergstrom	2014	0	0	#DIV/0!	VAS>7 (pain during pregnancy)					3,21	0.87-12.51				need adjusted estimate
Robinson	2010	179	176	98,3	LBB PRE-PREGN.					148,4	1.35-18,033	148,4	1.65-13,359	BMI, ASLR test	Betas, not OR, not %
Gausel	2015	309	36	11,7	LBP PRE-PREGN.	20	16	86	187	2,71	1.26-5.89	2,8	1.2-6.4	n=285, age and ODI	Only study without missing data
Albert	2001	341	29	8,5	Objective pain tests N>=16					10,7					describe media, no tests, no %
Robinson	2010	179	176	98,3	Provocation test 6<N<8					148,4	6.05-1,202,604	33,1	3.32-29,732	BMI only	Betas, not OR, not %

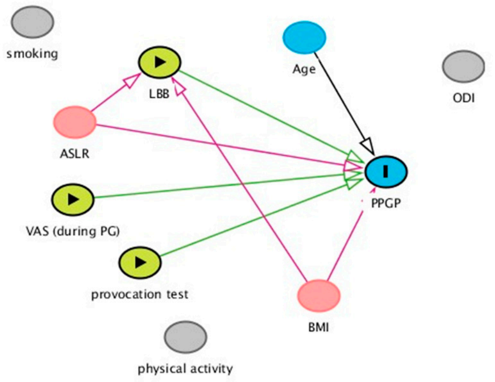

Data S4: *6 predictive factors infographic*

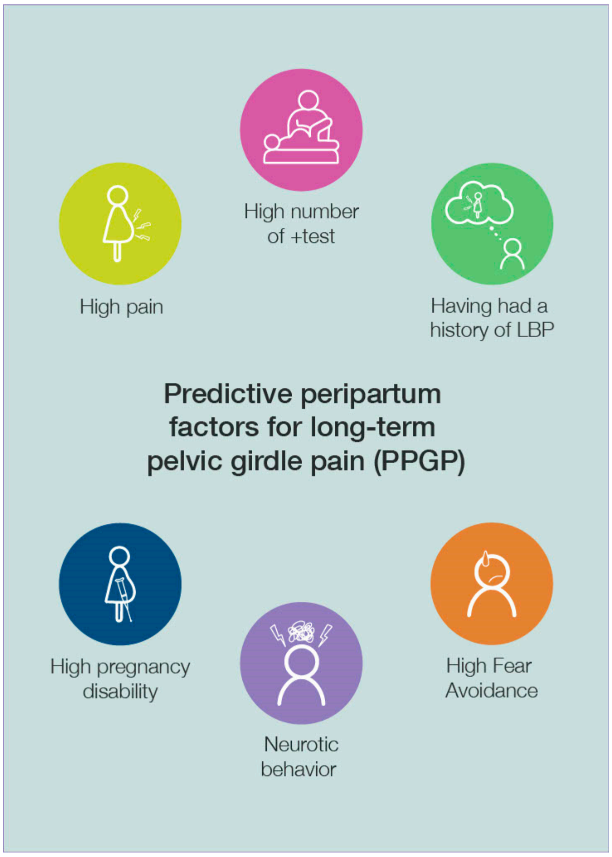

Supplement: Supplementary file 1 [file medicina-59-02123-s001.zip › medicina-2673293-supplementary.pdf]
